# Using amino acids co-occurrence matrices and explainability model to investigate patterns in dengue virus proteins

**DOI:** 10.1186/s12859-022-04597-y

**Published:** 2022-02-19

**Authors:** Leonardo R. Souza, Juan G. Colonna, Joseana M. Comodaro, Felipe G. Naveca

**Affiliations:** 1 Institute of Computing, Federal University of Amazonas, General Rodrigo Octavio Avenue, Manaus, Amazonas Brazil; 2 Institute of Biological Sciences, Federal University of Amazonas, General Rodrigo Octavio Avenue, Manaus, Amazonas Brazil; 3 Leonidas and Maria Deane Institute, Oswaldo Cruz Foundation, Terezina Street, Manaus, Amazonas Brazil

**Keywords:** Dengue virus, Protein amino acid co-occurrence, Machine learning

## Abstract

**Background:**

Dengue is a common vector-borne disease in tropical countries caused by the Dengue virus. This virus may trigger a disease with several symptoms like fever, headache, nausea, vomiting, and muscle pain. Indeed, dengue illness may also present more severe and life-threatening conditions like hemorrhagic fever and dengue shock syndrome. The causes that lead hosts to develop severe infections are multifactorial and not fully understood. However, it is hypothesized that different viral genome signatures may partially contribute to the disease outcome. Therefore, it is plausible to suggest that deeper DENV genetic information analysis may bring new clues about genetic markers linked to severe illness.

**Method:**

Pattern recognition in very long protein sequences is a challenge. To overcome this difficulty, we map protein chains onto matrix data structures that reveal patterns and allow us to classify dengue proteins associated with severe illness outcomes in human hosts. Our analysis uses co-occurrence of amino acids to build the matrices and Random Forests to classify them. We then interpret the classification model using SHAP Values to identify which amino acid co-occurrences increase the likelihood of severe outcomes.

**Results:**

We trained ten binary classifiers, one for each dengue virus protein sequence. We assessed the classifier performance through five metrics: PR-AUC, ROC-AUC, F1-score, Precision and Recall. The highest score on all metrics corresponds to the protein E with a 95% confidence interval. We also compared the means of the classification metrics using the *Tukey HSD* statistical test. In four of five metrics, protein E was statistically different from proteins M, NS1, NS2A, NS2B, NS3, NS4A, NS4B and NS5, showing that E markers has a greater chance to be associated with severe dengue. Furthermore, the amino acid co-occurrence matrix highlight pairs of amino acids within Domain 1 of E protein that may be associated with the classification result.

**Conclusion:**

We show the co-occurrence patterns of amino acids present in the protein sequences that most correlate with severe dengue. This evidence, used by the classification model and verified by statistical tests, mainly associates the E protein with the severe outcome of dengue in human hosts. In addition, we present information suggesting that patterns associated with such severe cases can be found mostly in Domain 1, inside protein E. Altogether, our results may aid in developing new treatments and being the target of debate on new theories regarding the infection caused by dengue in human hosts.

**Supplementary Information:**

The online version contains supplementary material available at 10.1186/s12859-022-04597-y.

## Background

Dengue is a viral infection that in most cases leads to a febrile syndrome without high clinical risk, accompanied by headaches, orbital, muscles and joints aches, nausea, vomiting and skin rashes. However, cases of severe dengue, like the dengue shock syndrome, occur with a certain frequency. Patients with severe dengue conditions may have difficulty breathing, severe bleeding, severe abdominal pain, frequent vomiting, fluid retention and fatigue. This combination of symptoms makes severe dengue potentially fatal [1]. Early identification of the infection combined with appropriate treatments can reduce the chances of fatality by more than 99% [2, 3].

Dengue cases may be found in all continents, excluding Antarctica. However, the virus has established and persisted endemically in urban areas of tropical and subtropical, which are favorable for the maintenance of the *Aedes aegypti* mosquito, the main vector of dengue [4, 5]. Statistical studies indicate that approximately 390 million people are infected every year, of which 96 million need some kind of medical attention [6]. Analyzes of infected patient samples indicate cases of fatal infection between 2.5% and 5.9% [7, 8]. Although the global distribution of dengue is uncertain, research indicates the establishment of the *Aedes aegypti* mosquito in 129 countries, suggesting a population of 3.9 billion people at risk for the infection [6, 9, 10].

Dengue viruses are divided into four groups of antigenically distinct serotypes, this feature enables dengue reinfections through new serotypes for the host’s immune system [11]. Despite this, it is believed that primary dengue infection generates heterotypic immunity within 1–3 years, and that secondary infection causes extensive cross-protection, resulting in rare post-secondary infections [12–14]. However, secondary infection increases the risk of severe dengue [15].

**Fig. 1:**
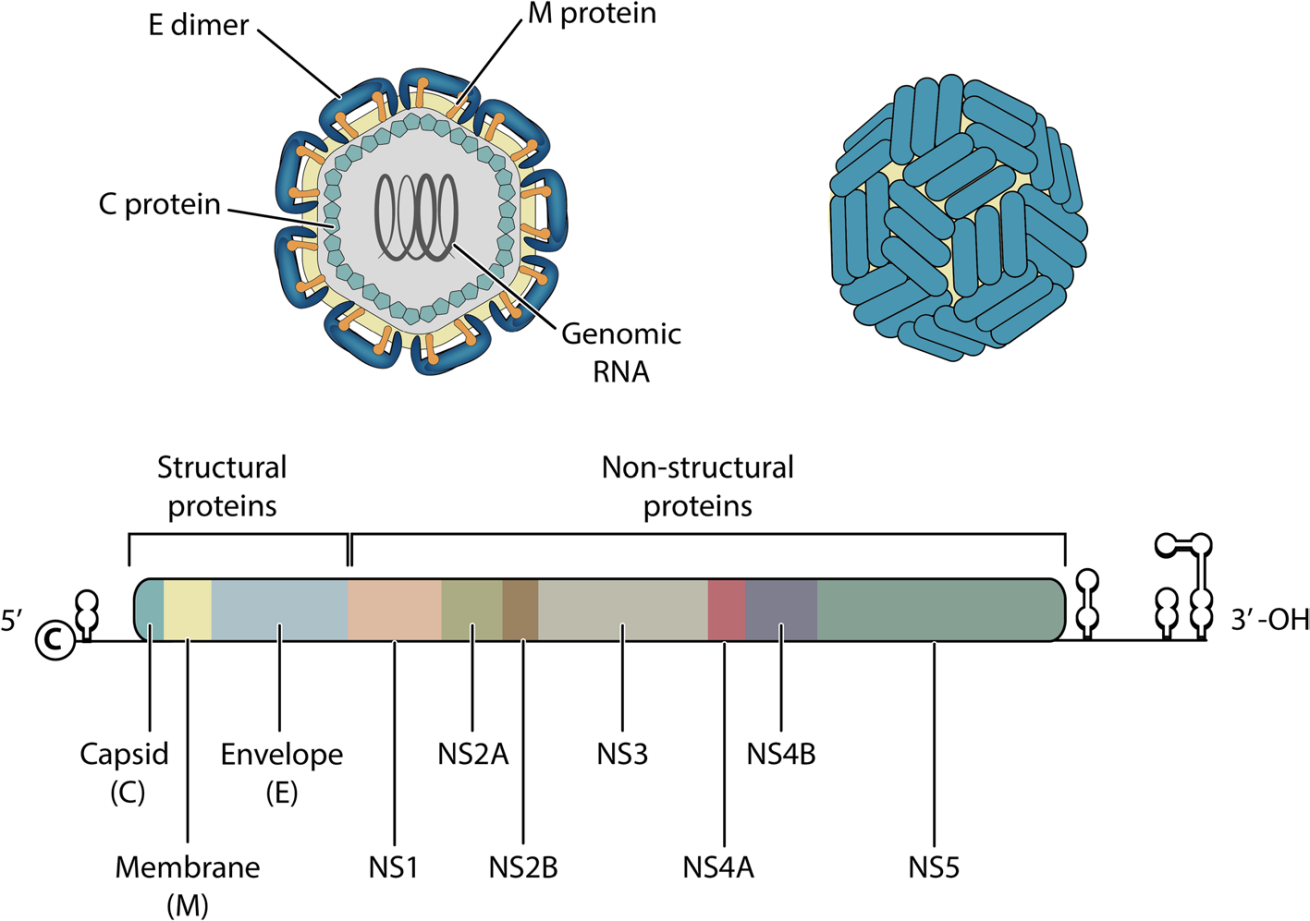
(Above) Artistic conception of a dengue virion. Genomic RNA is surrounded by structural proteins. (Below) Dengue virus RNA encoding representation. Each protein is indicated by a unique color. At the RNA ends it is possible to observe the regulatory regions 5’UTR and 3’UTR. As virion components, structural proteins work on viral entry, fusion and assembly, while non-structural proteins work on viral replication

The genetic material of the virus consists of single-stranded RNA (*single-stranded RNA*—ssRNA) with approximately 10,200 nucleotides and can be represented by a sequence of characters taken from a specific alphabet. To preserve the biological functions of each protein, specific relationships between nucleotides occur in every coding RNA. Thus, the dengue RNA coding region is divided into three structural proteins that make up the virion: C, M and E and; in seven non-structural proteins used in viral replication: NS1, NS2A, NS2B, NS3, NS4A, NS4B and NS5. In the complete genome illustrated in Fig. 1, the regulatory regions 5’UTR and 3’UTR that do not translate proteins, called ncRNA [16, 17] are also observable.

Each protein is responsible for a specific task. Protein E is responsible for recognition and entry into the cell to be infected [18], while protein NS1 participates of RNA replication and helps in the formation of immune complexes [19, 20]. The NS2A protein is important for viral pathogenesis, while the NS2B and NS3 proteins play an important role in viral protease functions [21–23]. The NS4A protein is associated with the M protein through internal regions and performs membrane rearrangement [24]. There is evidence that NS4A and NS4B can function cooperatively in viral replication and anti-host response [24, 25]. Finally, the NS5 protein bypasses the infected organism’s innate immune response system and is the viral RNA-dependent RNA polymerase [26, 27].

In this study, we explored and compared all these dengue proteins looking for amino acid patterns that may be associated with severe dengue. Machine learning algorithms rely on numerical inputs to perform prediction tasks. Based on this need, we propose the encoding of protein coding sequence in co-occurrence matrices of amino acids.

For this, we assembled a data set, in which the coding RNA sequences were aligned, translated and segmented to obtain the deduced proteins. We then encode these proteins into amino acid co-occurrence matrices, labeling them with the associated degrees of infection. Subsequently, these matrices are classified by a Random Forest (RF). Finally, the instance-label associations learned by the classifier are interpreted locally using SHAP Values (SHapley Additive exPlanations), revealing the co-occurrence patterns of amino acids that increase the probability of severe dengue in the sample.

Our results suggest that protein E has a better association with the degree of infection, with more relevant patterns for severity present in the region called Domain 1 of this protein. In addition to these results, the database of this work can be considered an additional contribution, as we provide data from protein-segmented dengue RNA samples containing information on the serotype and severity of the host-associated infection.

## Methods

### Framework for severe dengue explanation

The general objective of this research is to explore, through a machine learning (ML) explainability technique, the interaction between amino acids present in dengue proteins and how they generate patterns capable of associated the severity of dengue infection. For this, our framework is divided into 5 steps, namely: (1) viral RNA alignment and protein segmentation so that they can be explored independently; (2) sequence normalization and tokenization as steps to standardize and obtain protein amino acids; (3) generation of co-occurrence matrices of amino acids that will serve as training data for the classifier; (4) prediction of the degree of infection through the Random Forest (RF) algorithm and; (5) local explanation of the RF classification model for the training samples in order to extract sets of co-occurrences of significant amino acids for prediction of severe dengue.

### Input data

Proteins are chains of amino acids, such that amino acids are represented by characters taken from a specific alphabet known as IUPAC (International Union of Pure and Applied Chemistry) [28]. Let *P* be a protein such that, for any $p_i \in A$, *P* can be mathematically represented by the series $P = p_1p_2p_3 \ldots p_{n-1}p_n$, where $p_i$ is a amino acids, *A* is the alphabet and *n* is the number of amino acids in the protein.

**Fig. 2:**
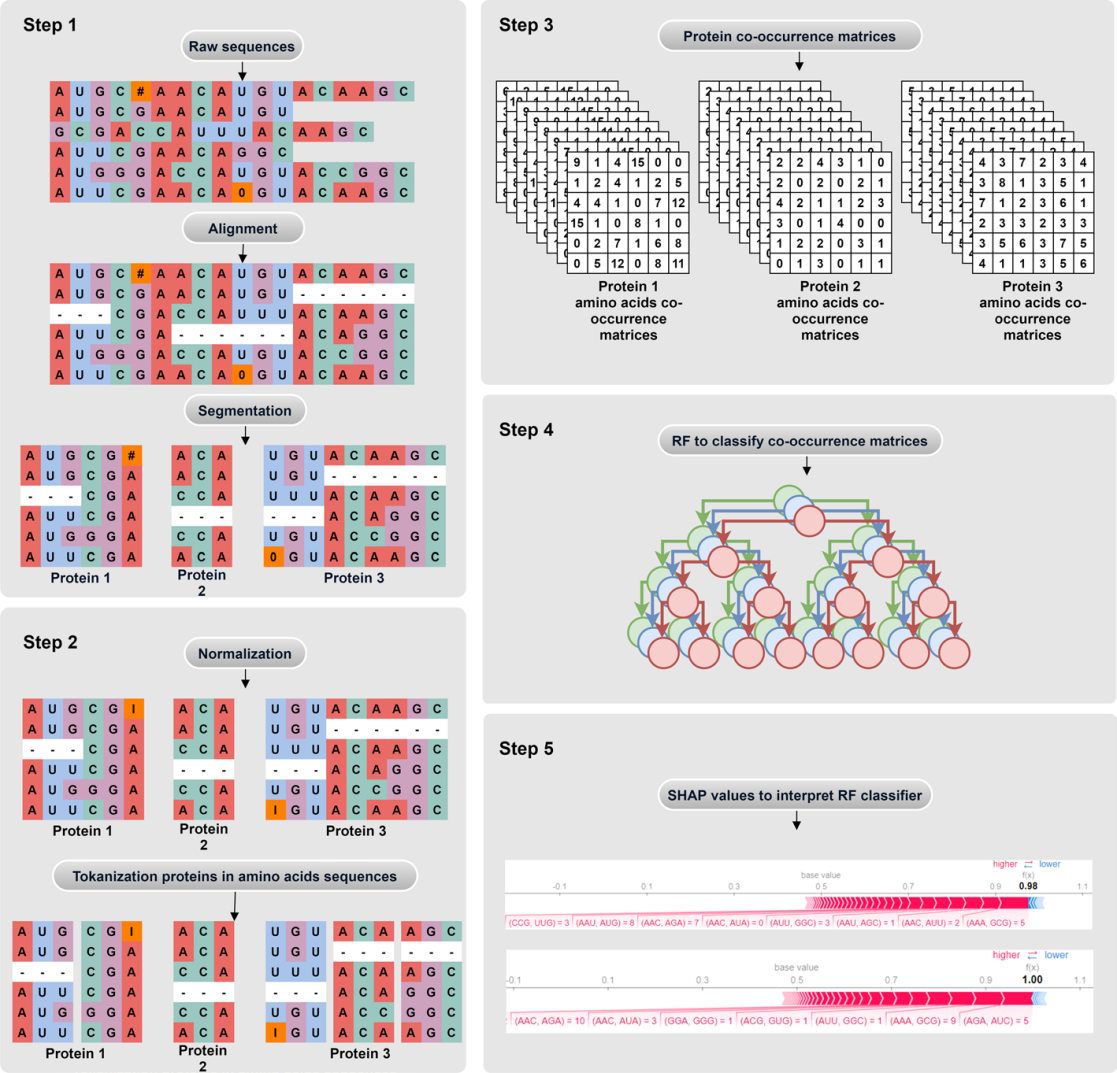
Methodology diagram. In the example, the method receives raw sequences containing proteins 1, 2 and 3 as input. Once aligned, it is possible to segment each protein. Then, the normalization and tokenization protein sequence processes are performed. Subsequently, amino acid co-occurrence matrix sets are generated for each protein, which will be classified by an individual RF for each protein. Finally, each RF is interpreted by Shap Values, thus generating explanations for each protein

### Data scraping

Despite the large amount of dengue genomes publicly available for research in gene sequence repositories, we found a great scarcity of samples labeled with the clinical picture of the infected patient. Therefore, we mine the NCBI (National Center for Biotechnology Information) and NCBI Virus Variation repositories in search of dengue genomic sequences labeled with the patient’s clinical outcome. A total of 562 labeled samples were obtained. Of this total, 61 samples have the complete dengue genome encoding all 10 proteins. For each protein, we generate a separate data file in the following order: Additional file 1: C protein, Additional file 2: M protein, Additional file 3: E protein, Additional file 4: NS1 protein, Additional file 5: NS2A protein, Additional file 6: NS2B protein, Additional file 7: NS3 protein, Additional file 8: NS4A protein, Additional file 9: NS4B protein, and Additional file 10: NS5 protein. This subset of carefully selected sequences is another a contribution of our work. We also make a copy available in a public repository via the link 10.5281/zenodo.5885637.

The labels found were: dengue fever (DF), dengue hemorrhagic fever (DHF) and dengue shock syndrome (DSS). Given the low amount of DHF and DSS samples and because they are severe cases of dengue, we performed the binary labeling of our database, where DF became “classic dengue” and DHF and DSS, “severe dengue”. All samples, with the exception of two samples collected from the spleen, were collected through blood material isolated from infected humans between 1985 and 2017. Data are from Brazil, Cambodia, Chile, China, Colombia, Cuba, Spain, Philippines, Ghana, India, Indonesia, Japan, Malaysia, Mexico, Paraguay, French Polynesia, Sri Lanka, Vietnam, Thailand and Taiwan (Republic of China).

### Protein sequences pre-processing

To avoid non-conformities in the classification and explanation of results steps, the protein sequences go through the steps of: alignment, normalization and tokenization, as illustrated in Fig. 2.

#### Sequence alignment and segmentation

The sequences were aligned using the MUSCLE algorithm available in the UGENE [29] software. MUSCLE is a three-stage alignment algorithm for multiple sequences [30]. After the alignment is completed, protein segmentation is performed. The segmentation of enconding sequences into deduced proteins was performed based on the reference sequences available in GenBank for each dengue virus serotype.

Sequence alignment allows for standardization of raw data samples, filling incomplete sequences with gaps so that they line up with 61 samples with complete genomes, allowing the creation of a database for each protein (Fig. 2). The sequence alignment process is based on the calculation of similarity of conserved regions between sequences. Therefore, it is natural that the alignment adds gaps in partially incomplete sequences so that the conserved regions of each sequence are aligned, increasing the similarity between sequences [30–32]. This procedure can result in extensive gap regions for very incomplete sequences, causing entire proteins to be represented solely by gaps. To get around this problem, before any processing to generate co-occurrence matrices, we chose to remove samples formed by more than 15% of gaps. For the remaining sequences, the gap character “-” was removed, since it has no meaning and was entered by the alignment algorithm. For instance, the sequence “- - - -ACAGAA- - - - -” becomes “ACAGAA”, while the sequences “ACA-GUA” and “ACA- -GUA” becomes “ACAGUA”.

The alignment, filling, selection and segmentation procedure ended up generating 10 databases, one for each protein. Furthermore, based on the hypothesis that identical samples could be used in several researches and that, moreover, duplicate samples do not add value to the learning of a ML classifier, identical sequences of the same coding protein were eliminated. After that, the final distribution of the bases can be seen in Table 1.

**Table 1:** Generated database distributions

Protein	Samples				
	Total	Duplicates	Remaining	Classic	Severe
C	298	192	106	71	35
M	288	155	133	91	42
E	394	123	271	190	81
NS1	275	118	157	110	47
NS2A	270	132	138	104	34
NS2B	270	167	103	70	33
NS3	270	88	182	132	50
NS4A	270	145	125	85	40
NS4B	270	144	126	89	37
NS5	270	60	210	148	62

#### Normalization

The normalization step consists of analyzing the nucleotides of the sequences, standardizing nucleotides without biological meaning, probably caused by sequencing errors. Therefore, in normalization, nucleotides that are not defined in the IUPAC nucleotide code are replaced by the pattern character I that represents indeterminacy.

#### Tokenization

Tokenization consists of segmenting each sequence into smaller subsequences, obtaining an ordered list of these subsegments. In our experiments, codons are the sequence substructure used for tokenization. Codons consist of nucleotide triplets that can be transcribed to amino acids [33]. Then, in the tokenization step, the amino acids of each protein sequence are obtained.

### Amino acid co-occurrence matrices

Co-occurrence matrices have been used to collect statistics from varied data, especially image and text data [34–36]. In medical image analysis, co-occurrence matrices are used to measure image textures [37]. In the field of Natural Language Processing (NLP), co-occurrences can provide clues to semantic relationships between words in a body of text [38]. The application of co-occurrence matrices also expands into the field of bioinformatics, for example, in protein sequences, evidence of important functional relationships for protein biological processes can be found when identical patterns of amino acid co-occurrence are present in different regions [39, 40].

A amino acid co-occurrence is the occurrence of two amino acid in a protein segment. Let *P* be a sequence of amino acid and *S* a segment of *P*, the co-occurrence matrix *X* can be obtained by the formula: $X_{ij} = \sum _{S} K_{ij }$, where,

1 $$\begin{aligned} K_{ij} = {\left\{ \begin{array}{ll} 1, &{} \text {if}\ i,j\in S \\ 0, &{} \text {otherwise} \end{array}\right. } \end{aligned}$$

and $X_{ij}$ denotes the number of times amino acid *j* was in the same segment as amino acid *i*. Thus, $X_{i,j}$ is proportional to the joint probability *P*(*i*, *j*), which represents the probability of occurrence of the terms *i* and *j* in the same segment.

The segment, or context window, reflects on the type of information provided by the matrices, for example, large segments reflect the coverage of large areas of the genome, generating co-occurrences between distant amino acid and reflecting on the ability of the co-occurrence matrices to capture long-distance correlations. Similarly, small segments define a search for closer patterns within a small region.

**Fig. 3:**
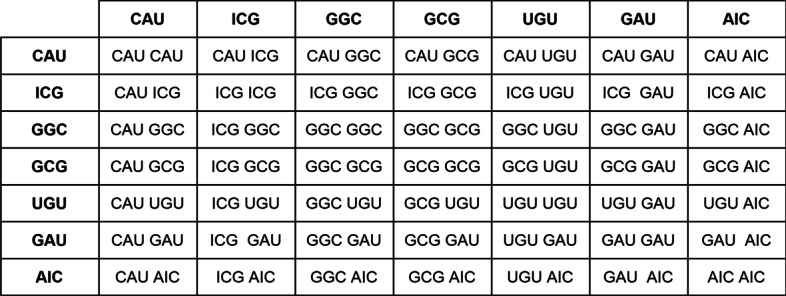
Co-occurrence matrix. A template matrix for samples $A_1$, $A_2$ and $A_3$

In order for the co-occurrence matrices of each sample of the same coding region to have identical dimensions, it was necessary to create a global dictionary containing all amino acids present in the samples. With possession of the global dictionary, it was possible to generate a template co-occurrence matrix that integrates all its co-occurrences. For example, let the samples be $A_1 = \lbrace {[CAU][ICG][GGC]\rbrace }$, $A_2 =\lbrace {[CAU][GCG][UGU]\rbrace }$ and $A_3 = \lbrace {[GAU][GCG][AIC]\rbrace }$ it is possible to get the global amino acid dictionary $d =$ {*CAU, ICG, GGC, GCG, UGU, GAU, AIC*} which allows us to generate the template co-occurrence matrix present in Fig. 3. The fact that co-occurrences are interchangeable generates a symmetrical co-occurrence matrix.

### Co-occurrence matrix resizing and vectorizing

Based on the symmetry of the co-occurrence matrices, the first scaling step is to extract only elements of the upper triangular matrix. The generated co-occurrence matrices have dimensions ${\mathbb {R}}^{d\times d}$, where *d* is the size of the amino acid dictionary. The fact that the matrices are symmetric and interchangeable allows the resizing of the upper triangular matrix into a vector of dimension ${\mathbb {R}}^{d(d+1)/2 \times 1 }$. Finally, through these vectors it is possible to build a tabular database, where each column of the base represents a co-occurrence between pairs of amino acids.

### Feature selection

In order to achieve maximum classifier performance by reducing problem complexity and eventually an overfitting, we eliminate co-occurrences that carry little or no information. For this, we use the Mutual Information (MI) algorithm that measures the dependence between two variables by calculating entropy using the k-nearest neighbors. In this context, two variables can be considered independent if, and only if, the MI coefficient between them is zero. In contrast, the greater the dependence between two variables, the greater their mutual information value [41, 42]. Therefore, mutual information values between co-occurrences and clinical picture were calculated for each protein base. Finally, the 50 co-occurrences that presented the greatest mutual information related to the clinical picture of dengue were selected for each database.

### Random-forest

**Fig. 4:**
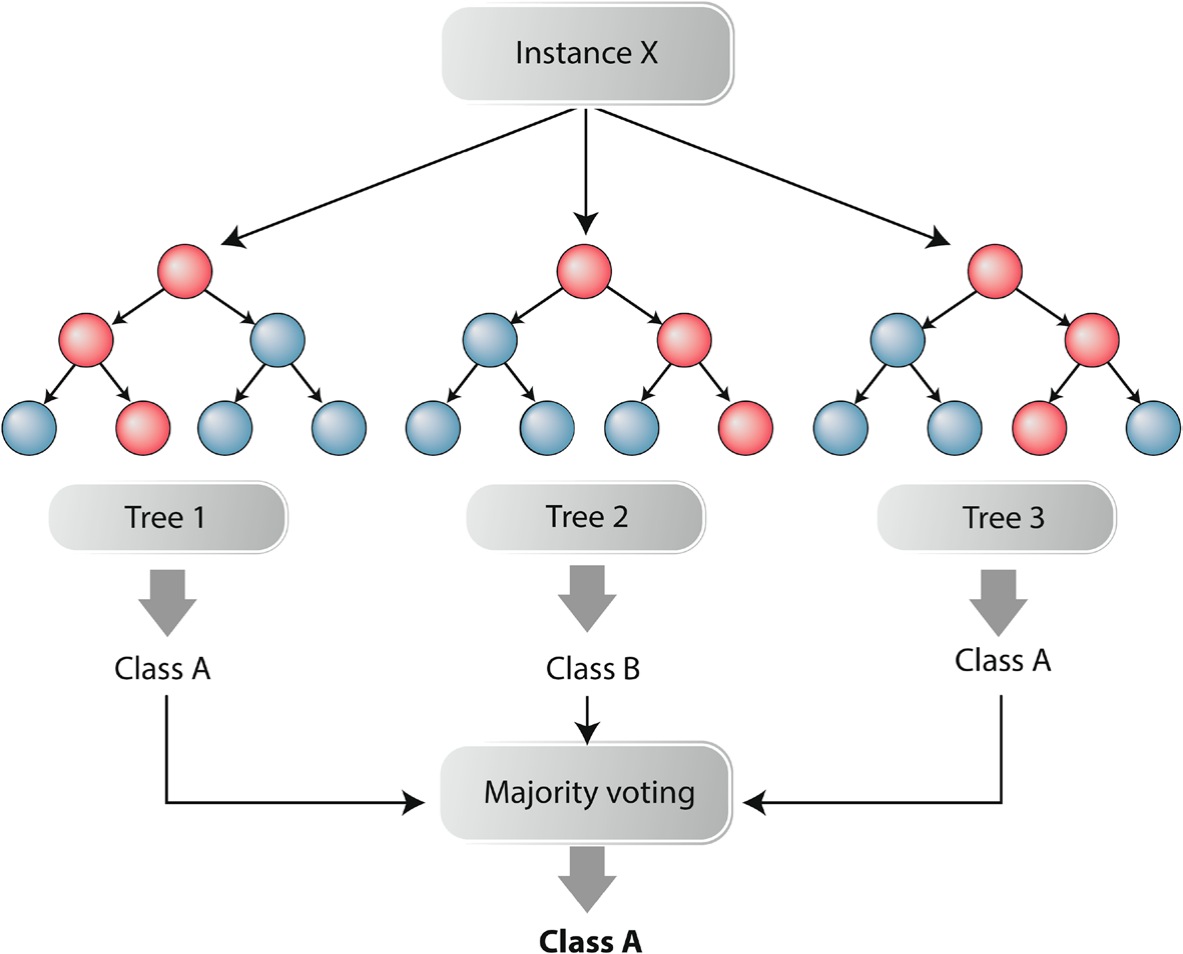
Random-forests example. Three decision trees compose the RF classifier. The red nodes represent the path taken by the data until the classification leaf

The scarcity of publicly available samples with the clinical outcomes makes complex classification algorithms like CNN and LSTM have great difficulties in learning patterns in our data, considering the large amount of samples that these algorithms require for parameter optimization. Therefore, we chose to use the Random-Forest (RF) classifier for our experiments. Overall, RF classifiers are significantly less complex than *deep* machine learning methods, yet they are still widely used in the field of bioinformatics [43–47]. RF (Fig. 4) can be defined as models that consist of structured collections of $\lbrace {h(x, \Theta _k), k=1, \ldots \rbrace }$ decision trees , where $\Theta _k$ are independent and identically distributed variables and *x* is an input vector. After generating the trees, RF selects the most popular class among the trees for input *x* [48].

The RFs are part of a set of methods called *ensembles*, which are nothing more than combinations of several models to obtain a single result, making the *ensembles* more robust when compared to simpler algorithms such as trees decision or kNN [49, 50]. The basic structure of RF have as their basic unit binary decision trees (binary estimators) that employ recursive data partitioning.

To build each decision tree, the algorithm randomly selects variables from the training data and, from these, selects the most informative one to be the initial node (root node) that will have the first condition verified, giving rise to two child nodes that will initiate branches to the left and right of the root node. The node generation process is repeated throughout the tree, determining rules that define the data flow through the tree’s branches and establish its decision making [43, 51]. All these processes are repeated in the generation of the next trees. Finally, the RF defines the predicted class based on the class vote of the generated n-trees, where, the most predicted class in all the trees will be the final class of the RF [43].

### Model explainability with SHAP Values

Many machine learning algorithms are considered functional black boxes because, given their complexity, it is almost impossible to understand their internal processes. However, in bioinformatics it is essential that there is a human domain over the classifier’s decisions. Given this issue, several explainability methods have been proposed to explore the decisions made by ML models by evaluating the influence of input variables on the prediction results [52–56].

We can also mention other explanation techniques used in biological sequence classification problems through Deep Learning (DL) models [57–59], where the classifier is a Convolution Neural Network (CNN). Therefore, in these works it is assumed that the explanations are linked to the significant values of the CNN filters and the positions in which these values occur, then these values are backtracked to the input sequence and the relevant patterns are collected. As they are DL-based models, they need large amounts of data to be trained and explained, and unfortunately, our small amount of samples makes it impossible to use DL-based methods. Therefore, given the limitations imposed by the amount of samples, we chose the Random Forest classifier and used the SHAP values method with its specific explainer for tree-based models.

Explainability methods are divided into two classes: global methods that explain model results for all data inputs; and local methods that explain an individual input. Our interest in model explanation is to be able to understand what happens in the classification of severe dengue, making it possible to identify significant amino acids co-occurrences for classifier assign a sample to the severe dengue class. Therefore, in explaining the model we want to encode its learned patterns and decision-making into information explainable in human terms.

Therefore, we decided to use in our experiments the SHAP Values [54] method that performs a local explanation under the trained model and the instance of interest, making it possible to independently interpret classical dengue samples and severe dengue samples. The basic concept of SHAP Values is to ensure that two models *f* and *g* have approximate results for each instance. For this to occur, the condition $g(x^{\prime }) - f(h_x(x^{\prime })))$, where *f* is the original predictive model, *g* is the interpreter model, and $x^{\prime }$ is a simplification of the original instance *x* that can be mapped to the original instance from a function *h*, such that $x = h_x(x^{\prime })$. For a more detailed understanding, SHAP Values unifies the importance of variables through a conditional expected value function of the *f* model, such that, $f_x(z^{\prime }) = f(h_x(x^{\prime ) })) = E(f(x)|x_S)$, where *S* is the non-null subset of $x^{\prime }$. Finally, the general equation of the method explanation model takes the form of the conditional expectation function $f(h_x(x^{\prime })) = E(f(x)|x_S)$ [54].

#### TreeExplainers

TreeExplainer is a specific method for local explanations of tree-based models, providing fast and accurate results by calculating the SHAP values for each leaf of a tree. The algorithms estimate $f(h_x(x^{\prime })) = E(f(x)|x_S)$ recursively following the decision path for an input instance *x* in a tree. The complete methodology, as well as the algorithms that define the TreeExplainer, can be found at [60].

#### SHAP Values explanations results

Machine learning models internally perform multiple mathematical operations to obtain results. For example, to perform predictions, classifiers generate real values which in turn will be associated with labels. As described earlier, SHAP Values performs variable explanation from the conditional expectation function.

From there, the method assigns positive and negative impacts to the input instance variables so that the expected value of the interpreter $E(f(x)|x_S)$ is equal to the output value of the original model *f*. Thus, the magnitude of the impact reflects the influence of the variable in the classification of the sample, such that positive impacts increase the probability of correct classification of the sample, while negative impacts have the opposite effect, suggesting that variables with positive impacts have a greater capacity to characterize the sample class [61]. Therefore, for each sample, the SHAP Values method generates a table that associates a classification impact value with the features in the sample.

To facilitate viewing the patterns provided by SHAP, we chose to generate a global explanation from multiple local explanations. For this purpose, after obtaining all the tables, the positive impact score of each co-occurrence is calculated, which consists of the number of times each co-occurrence had a positive impact divided by the number of times the co-occurrence appeared. Then, the average impact value of each of them is calculated. After that, each co-occurrence is ranked in descending order by the two metrics. Finally, we selected the resulting co-occurrences located in the first 20% ranking positions and the final 20%. That is, the 20% with the highest positive impact and the highest positive impact score and the 20% with the lowest positive impact and lowest positive impact score.

### Experiments and results

**Fig. 5:**
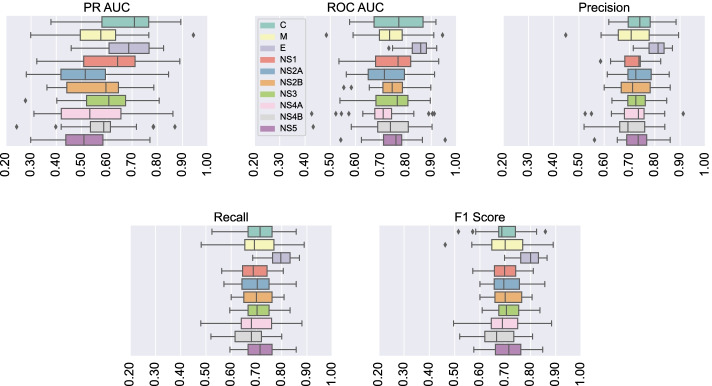
Comparison of results. Proteins M, NS1, NS2A, NS2B and NS4A show results with greater variability in the cross-validation fivefolds, while proteins C, E, NS4B and NS5 indicate less variability. For the recall and F1-score metrics, the NS3 protein showed low variability, however, containing outliers. The distribution of protein E metrics gives evidence that its results are superior. Furthermore, the distribution of E protein metrics show low variability, showing that the classifier maintained a uniform performance for each fold

Five stratified cross-validations were performed to observe the classifier’s response on different training and test sets. In view of the evident unbalance of classes in the bases presented in the Table 1, the PR-AUC metric (Area Under the Precision-Recall Curve) [62] was chosen to evaluate the model, in addition to the metrics: ROC-AUC metric (Area Under the ROC Curve), precision, recall and balanced F1-score. Precision, recall, and F1-score balanced metrics compensate for class imbalance by calculating a weighted average across correctly classified instances, while ROC-AUC is more optimistic than PR-AUC for unbalanced datasets. The mean of the metrics, as well as their confidence intervals for all proteins can be seen in Table 2. Also, we perform exploratory analyzes to observe the classifier performance in each database. To visually compare the results obtained for each database, we used box-plots (Fig. 5) to verify the empirical distribution of the metrics.

**Table 2:** Average of the metrics obtained in fivefolds cross validation with 95% of confidence interval using Student’s t-distribution

Protein	PR-AUC	ROC-AUC	P	R	F1
	*Mean* ± *E*	*Mean* ± *E*	*Mean* ± *E*	*Mean* ± *E*	*Mean* ± *E*
C	$0.66 \pm 0.06$	$0.77 \pm 0.04$	$0.74 \pm 0.03$	$0.70 \pm 0.03$	$0.70 \pm 0.03$
M	$0.57 \pm 0.06$	$0.73 \pm 0.04$	$0.71 \pm 0.03$	$0.70 \pm 0.03$	$0.70 \pm 0.03$
E	$\mathbf{0.67 } \pm \mathbf{0.04 }$	$\mathbf{0.85 } \pm \mathbf{0.02 }$	$\mathbf{0.80 } \pm \mathbf{0.02 }$	$\mathbf{0.79 } \pm \mathbf{0.02 }$	$\mathbf{0.79 } \pm \mathbf{0.02 }$
NS1	$0.62 \pm 0.06$	$0.74 \pm 0.04$	$0.72 \pm 0.02$	$0.69 \pm 0.03$	$0.70 \pm 0.03$
NS2A	$0.52 \pm 0.06$	$0.73 \pm 0.04$	$0.74 \pm 0.02$	$0.70 \pm 0.03$	$0.71 \pm 0.03$
NS2B	$0.57 \pm 0.05$	$0.74 \pm 0.03$	$0.73 \pm 0.03$	$0.70 \pm 0.03$	$0.70 \pm 0.03$
NS3	$0.58 \pm 0.05$	$0.75 \pm 0.04$	$0.73 \pm 0.02$	$0.71 \pm 0.02$	$0.71 \pm 0.02$
NS4A	$0.55 \pm 0.06$	$0.71 \pm 0.05$	$0.72 \pm 0.04$	$0.69 \pm 0.04$	$0.69 \pm 0.04$
NS4B	$0.58 \pm 0.05$	$0.74 \pm 0.04$	$0.70 \pm 0.03$	$0.67 \pm 0.03$	$0.68 \pm 0.03$
NS5	$0.52 \pm 0.05$	$0.75 \pm 0.03$	$0.73 \pm 0.03$	$0.71 \pm 0.03$	$0.71 \pm 0.03$

Highlight the best results obtained through bold text

The P, R and F1 columns represent the Precision, Recall and F1-Score metrics, respectively

It is possible to observe in the box-plots in Fig. 5 that for the fivefolds of validation, the results of each metric for proteins M, NS1 ,NS2A and NS4A have a high variance when compared to the other proteins. On the other hand, the box-plots of protein E have low variance in Precision, Recall and F1-score metrics, indicating that for each fold the results obtained are more constant than in the other proteins, which suggests a greater capacity for generalization by the classifier when it uses protein E data.

Furthermore, the box-plots of the Precision, Recall and F1-score metrics in Fig. 5 show a possible difference between the results obtained for each protein. Therefore, to statistically test the hypothesis that the mean results are different for each protein, we used the one-way analysis of variance (ANOVA) model, which compares sample means through the Fisher-Snedecor F distribution [63, 64]. The ANOVA test hypotheses are: the null hypothesis $H_0$, where the sample means are equal, and the alternative hypothesis $H_1$, where at least one of the averages is different from the others.

The data used in the ANOVA test must meet the assumption of homogeneity of variances, verified by the Levene test [65], as well as the model’s residuals must be normally distributed, verified by the Shapiro–Wilk test [66]. The null ($H_0$) and alternative ($H_1$) hypotheses for Levene’s test are: the groups variances are homogeneous and the groups variances are not homogeneous, respectively. For the Shapiro–Wilk test the hypotheses are: $H_0$ data is normally distributed and $H_1$: data is not normally distributed. All null hypotheses are accepted if, and only if, the p-value of the test is greater than a significance level of $\epsilon$. The Table 3 presents the results of the ANOVA tests for each metric, as well as the tests of their assumptions.

**Table 3:** For a significance level of $\epsilon = 0.05$ the Levene and Shapiro–Wilk tests show evidence that the metrics have homogeneous variances and that the residuals of the ANOVA model are normally distributed

	Leven’s test *p* value	ANOVA *p* value	Shapiro–Wilk *p* value
PR-AUC	0.83	$4\times 10^{-5}$	0.83
ROC-AUC	0.10	$6\times 10^-5$	0.09
Precision	0.07	$4\times 10^{-4}$	0.43
Recall	0.11	$2\times 10^{-5}$	0.53
F1-score	0.13	$2\times 10^{-5}$	0.63

Finally, the null hypothesis of the ANOVA test is rejected, indicating that at least one of the metric means is different from the others

**Fig. 6:**
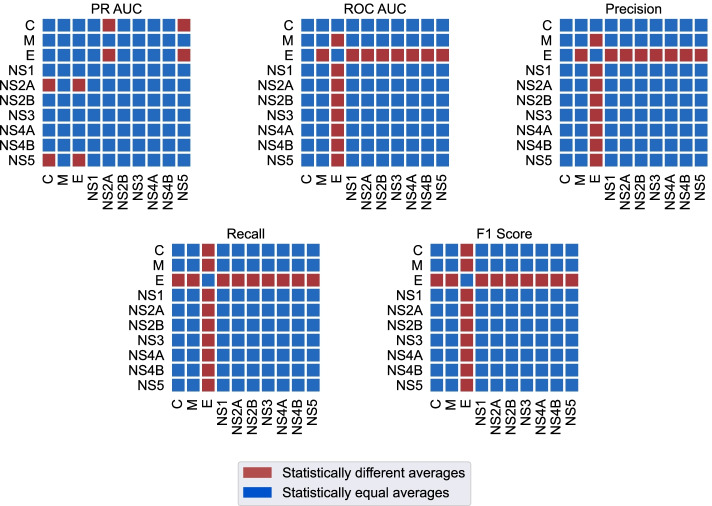
For the Tukey test with a significance level of $\epsilon = 0.05$, protein E metrics were statistically different from other proteins, with the exception of PR-AUC and Precision, which were statistically equal to protein C. In these experiments, the co-occurrences present in protein E have a greater capacity to describe the severity of dengue when compared to other proteins

After obtaining the confirmations of the ANOVA test, we applied the Tukey test to verify the difference between the means of the metrics for each protein. The null hypothesis for Tukey’s test assumes that there is no statistically significant difference between the means of two samples, while the alternative hypothesis assumes the opposite. Protein pairs with statistically distinct means of metrics can be seen in Fig. 6. As we can see, for all metrics, protein E presents statistically different averages at least one protein in Tukey pair comparison.

### Explanations

After being trained, the classifiers were interpreted using the SHAP Values method through the *TreeExplainer* algorithm. The SHAP Values method generates individual explanations for each data sample. For our explanations we use force plots, which in turn show the impact of sample variables on the prediction [61]. Then, from the force plots we can extract the impact of each co-occurrence on the probability of classification of severe dengue. Therefore, the first step of our explanations is to rank the co-occurrences that increase the probability of severe dengue, so that, finally, we can visualize the distribution of these co-occurrences and their behavior in samples of classic dengue.

Of the 50 co-occurrences selected by the MI algorithm, the explanation graphs will be 20% of the most relevant co-occurrences in the classification of severe dengue and the 20% less relevant. Finally, the co-occurrence values will be compared with classic dengue samples. As stated earlier, explanations generate positive and negative impacts. Co-occurrences do not have a constant impact behavior for each sample, that is, the same co-occurrence may have positive impacts in certain samples and negative impacts in severe dengue samples.

#### E protein explanations plots

**Fig. 7:**
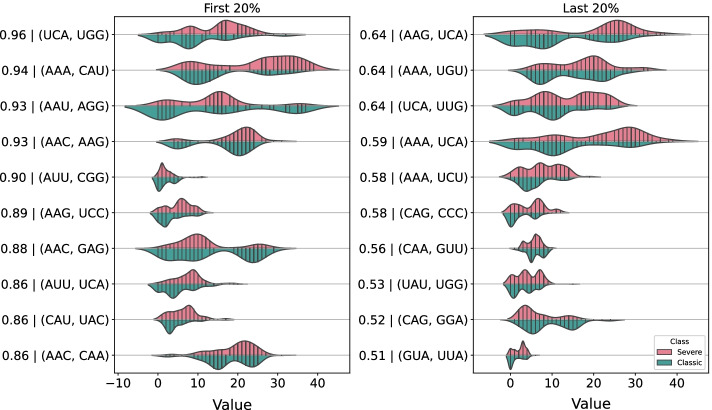
The figure shows the density graphs of the co-occurrence distributions that were interpreted as significant for severe dengue (in pink) and, for comparison purposes, their density for classic dengue (in green). Each label on the *y* axis is composed of a probability followed by the co-occurrence, for example, for the First 20% the first co-occurrence (UCA, UGG) positively impacted $96\%$ of the severe dengue samples , that is, the probability of severe dengue increased in $96\%$ of the samples. The *x* axis contains the co-occurrence values.

Protein E explanations reveal distinct characteristics between co-occurrences of significant amino acids for severe dengue compared to classic dengue. In general, as we can see in Fig. 7, the co-occurrence distributions are mostly distinct for classic and severe dengue. Examining the Fig. 7 we can observe differences in the behavior of the empirical distributions of amino acids significant for severe dengue compared with their behavior in classic dengue. These differences are more evident for the co-occurrence between the amino acids Serine and Tryptophan (encoded by UCA and UGG, respectively) which is positively significant in 96% of severe dengue samples. In this we can observe that the value distribution of this co-occurrence tends to have higher concentrations, close to 10, while for severe dengue this figure rises to 20.

We can observe that for all cases the empirical distributions of significant co-occurrences for severe dengue are not graphically identical to those for classic dengue, although they are close in some cases. Again, it is important to emphasize that the co-occurrences present in Fig. 7 are ranked according to their importance in the classification of severe dengue in the samples. For example, the first co-occurrence (UCA, UGG) was significant for classification of 96% of severe dengue samples, while the last co-occurrence (AAG, CGC) was significant for classification of only 35% of severe dengue samples.

#### Co-occurrences importance by E protein regions

Dengue E protein can be divided into four major regions, namely: Domain 1, Domain 2, Transmembrane 1 and Transmembrane 2. Each of the four dengue serotypes have specific RNA positions that mark the beginning and end of these regions [67–72]. To improve the visualization, after analyzing the behavior of the co-occurrences for samples of each serotype, the co-occurrence values by region for samples of each serotype are grouped through the mean, as can be seen in Table 4.

**Table 4:** Domain 1 has on average more significant co-occurrences for severe dengue

Co-occurrence	Domain 1	Domain 2	Transmembrane 1	Transmembrane 2
(UCA, UGG)	**4.33**	1.81	0.79	0.06
(AAA, CAU)	**10.30**	3.07	0.03	0.42
(AAU, AGG)	**8.30**	0.48	1.28	0.22
(AAC, AAG)	**8.98**	3.45	0.00	0.00
(AUU, CGG)	**1.40**	0.06	0.34	0.00
(AAG, UCC)	**1.22**	1.06	0.03	0.00
(AAC, GAG)	**6.12**	4.06	0.00	0.00
(AUU, UCA)	**2.51**	1.40	0.66	0.03
(CAU, UAC)	**2.78**	1.52	0.00	0.00
(AAC, CAA)	**10.19**	3.35	0.00	0.00
(AAG, UCA)	**5.51**	3.51	0.04	0.00
(AAA, UGU)	**8.94**	2.12	0.00	0.00
(UCA, UUG)	**3.89**	1.64	0.46	0.12
(AAA, UCA)	**5.69**	4.37	0.08	0.81
(AAA, UCU)	**2.75**	1.15	0.12	0.06
(CAG, CCC)	**1.72**	0.32	0.31	0.00
(CAA, GUU)	**3.16**	0.79	0.00	0.00
(UAU, UGG)	**0.85**	0.07	0.12	0.27
(CAG, GGA)	**9.05**	0.69	0.01	0.00
(GUA, UUA)	0.61	**0.76**	0.12	0.00

For E protein regions, highlight, through the bold text, the highest average value of co-occurrences for the significant amino acid pairs

The Domain 1 region of dengue E protein has the highest mean concentration of significant co-occurrences for the classification of severe dengue. With the exception of the co-occurrence (GUA, UAA) which is on average more present in Domain 2, all the others are more frequent in Domain 1, as we can see in Table 4. This is an indication that domain 1 may be directly related to the probability of dengue fever in the clinical outcome. However, more in-depth experiments are needed to confirm this evidence.

## Discussion

In this article, we present a method capable of representing and classifying severe dengue according to the protein coding sequence of the virus. Furthermore, the method is focused on improving the extraction of significant patterns for the classifier. The procedure is based on the segmentation of dengue viral RNA in each of the ten protein coding sequences, transforming these protein segments into matrices of co-occurrence of amino acids within a context window that will be classified by a RF.

The significant co-occurrences for severe dengue class were obtained through the SHAP Values explanation model, which employs a range of strategies to select variables that have greater weight in the classifier’s decision making, that is, co-occurrences that increase the probability of severe dengue. An important piece of information is that the context window is not automatically generated, this allows one to adjust the range of co-occurrences, allowing one to choose between performing local analyses, represented by patterns of co-occurrences conserved within the genome, or analyzes in large segments, allowing for co-occurrences between distant amino acids to be captured, increasing the chance of collecting long-distance correlations between amino acids.

Another important point to highlight is that by applying a classifier with few hyper parameters for adjustment, we reduce the need to use large databases for classification. Therefore, our method is able to perform on small databases, however, this does not mean that additional strategies are excluded, in our problem, for example, it was necessary to binarize labels to reduce the negative effects of high unbalance of our base. One of the advantages of using an RF as a classifier is that, because it is a rule-based classifier, the significant patterns for classification obtained by the SHAP method tend to be more concrete, since this classifier does not employ transformations in the input data, as with the deep models CNN and LSTM [73].

Finally, we emphasize that the focus of our approach is the exploratory analysis of the RNA sequences that produced a clinical outcome known as dengue severe, showing amino acid patterns that were related to this event. The presented methodology is flexible, as it would be possible to add metadata along with the co-occurrence vectors, such as mass, volume, polarity and charge of the protein segment. There are no limitations on the use of our method for classifying and interpreting other biological sequences.

## Conclusion

In this work, we described an ML method capable of identifying amino acid co-occurence patterns associated with severe dengue cases. In our analysis, precisely the same amino acids didn’t need to be found in all cases, but a signature of them. The biological basis of these results needs further evaluation, and other multifactorial aspects linked to dengue severe cases like secondary infection and host immunogenetics must not be ruled out. On the other hand, the method may be used as an interesting approach to identify patterns that may not be easily identified using other techniques.Moreover, the statistical analysis results do not support that the presented results occurred only by chance. Notwithstanding, the paucity of genomes with available outcome metadata may limit the robustness of some of the observed associations. Furthermore, we believe that the method described here may also be helpful for other studies with different viral agents.

## Supplementary Information

**Additional file 1.** C protein: A list of sequences belonging to Dengue virus protein C in csvformat. Each row is a sample of the amino acid chain labeledaccording to dengue severity.

**Additional file 2.** M protein: A list of sequences belonging to Dengue virus protein M in csvformat. Each row is a sample of the amino acid chain labeledaccording to dengue severity.

**Additional file 3.** E protein: A list of sequences belonging to Dengue virus protein E in csvformat. Each row is a sample of the amino acid chain labeledaccording to dengue severity.

**Additional file 4.** NS1 protein: A list of sequences belonging to Dengue virus protein NS1 in csvformat. Each row is a sample of the amino acid chain labeledaccording to dengue severity.

**Additional file 5.** NS2A protein: A list of sequences belonging to Dengue virus protein NS2A in csvformat. Each row is a sample of the amino acid chain labeledaccording to dengue severity.

**Additional file 6.** NS2B protein: A list of sequences belonging to Dengue virus protein NS2B in csvformat. Each row is a sample of the amino acid chain labeledaccording to dengue severity.

**Additional file 7.** NS3 protein: A list of sequences belonging to Dengue virus protein NS3 in csvformat. Each row is a sample of the amino acid chain labeledaccording to dengue severity

**Additional file 8.** NS4A protein:A list of sequences belonging to Dengue virus protein NS4A in csvformat. Each row is a sample of the amino acid chain labeledaccording to dengue severity.

**Additional file 9.** NS4B protein: A list of sequences belonging to Dengue virus protein NS4B in csvformat. Each row is a sample of the amino acid chain labeledaccording to dengue severity.

**Additional file 10.** NS5 protein: A list of sequences belonging to Dengue virus protein NS5 in csvformat. Each row is a sample of the amino acid chain labeledaccording to dengue severity.

## Data Availability

The datasets generated and/or analysed during the current study are available in the Github repository, https://doi.org/10.5281/zenodo.5885637
